# The relationship between sports facility accessibility and physical activity among Korean adults

**DOI:** 10.1186/s12889-016-3574-z

**Published:** 2016-08-26

**Authors:** Sang Ah Lee, Yeong Jun Ju, Joo Eun Lee, In Sun Hyun, Jin Young Nam, Kyu-Tae Han, Eun-Cheol Park

**Affiliations:** 1 Department of Public Health, Graduate School, Yonsei University, Seoul, Republic of Korea; 2 Institute of Health Services Research, Yonsei University, Seoul, Republic of Korea; 3 Department of Preventive Medicine and Institute of Health Services Research, Yonsei University College of Medicine, 50 Yonsei-ro, Seodaemun-gu, Seoul, 120-752 Republic of Korea

**Keywords:** Physical activity, Accessibility, Depression

## Abstract

**Background:**

The benefits of physical activity on physical and mental health are well known. The accessibility of sports facilities is reported to have considerable association with the amount of physical activity a person participates in. Therefore, we investigated the association between subjectively assessed accessibility of sports facilities and physical activity among Korean adults.

**Methods:**

We obtained data from the 2012 Community Health Survey. Physical activity was measured based on weekly metabolic equivalent task (MET) hours according to the International Physical Activity Questionnaire (IPAQ). Sociodemographic, economic, and health variables were used as covariates in a logistic regression model.

**Results:**

A total 201,723 participants were included in this study. Participants with easy access to sports facilities participated in physical activity more often than those without easy access (OR = 1.16, 95 % CI 1.13–1.20). More physical activity was generally observed if participants had a history of depression or if participants were among the white-collar or urban subgroups.

**Conclusion:**

Our results showed that the accessibility of sports facilities is associated with physical activity. Therefore, it is crucial to consider the accessibility of sports facilities when promoting an environment conducive to physical activity or designing programs for enhancing physical activity.

## Background

The health benefits of physical activity are well known throughout the world. Participating in regular physical activity declines mortality and has positive effect on chronic diseases, such as cardiovascular disease, hypertension, and diabetes mellitus [1]. Additionally, physical activity has a beneficial effect on mental diseases such as anxiety or depression [2]. It reduces stress and depression, and increases self-confidence and emotional well-being [3]. Thus, promoting physical activity is an important part of enhancing public health.

Despite these numerous benefits, physical activity levels have declined in the developed and developing countries [4]. Members of vulnerable social groups have an especially increased incidence of participating in unhealthy behaviors, including physical inactivity [5]. Additionally, those with lower levels of education or low economic status participate in physical activity less often compared to those of higher education and economic status [6]. The high rate of physical inactivity among these groups can cause significant public health problems. In addition to these groups, most people who suffer from depression also do not engage in physical activity compared to the general population [7]. According to the Epidemiology Survey of Mental Disorders in Korea 2011, the prevalence of depression is on the rise compared to other mental disorders. Additionally, lifetime prevalence and 1 year prevalence were almost 20 % higher in 2011 compared to 2006 [8]. Thus, identifying effective interventions to relieve this alarming depression rate is imperative. Considering the various benefits of physical activity, promoting physical activity may prove to be a successful intervention for alleviating depression and enhancing public health. In order to promote physical activity rates, identifying the factors associated with increased physical activity is essential.

Several determinants of physical activity have been reported. Whether or not a person participates in physical activity is affected not only by individual lifestyles, but also by the environment in which a person lives or works [9, 10]. Generally, the determinants of physical activity are categorized into five factors according to the ecological model: individual, interpersonal, environmental, regional or national policy, and global factors [11]. Among these five factors, environmental factors include three dimensions: social environment, built environment, and natural environment. Ecological models stand on the basis that physical activity is conducted at the particular physical environments such as sports facilities, and these places which are designed for physical activity could have an influence on the choice of physical activity [12]. In fact, the accessibility of sports facilities (an environmental factor) has been reported in several studies to dramatically affect individual participation in physical activity [13–16]. It is believed that the presence of sports facilities is associated with participation in physical activity [14, 16–18]. Particularly, closer distances between an individual’s home and a sports facility are associated with high levels of physical activity [19]. Therefore, many countries have been investing in sports facilities over the recent decades to promote physical activity [20].

Many previous studies reporting the association of the accessibility of sports facilities with physical activity have focused solely on specific generational groups, such as adolescents, college students, and seniors [13, 21, 22]. However, considering the high depression rate in South Korea and the beneficial effects of physical activity on depression, it is necessary to determine the relationship between accessibility of sports facilities and physical activity among depressed individuals. To our knowledge, there is no study exploring the determinants of physical activity among people with history of depression, particularly in the context of the accessibility of sports facilities. Additionally, there are no studies evaluating the association of the accessibility of sports facilities with physical activity among people of diverse sociodemographic groups.

Therefore, using nationally collected data, we analyzed the association between the accessibility of sports facilities and physical activity among individuals 20 years of age or older in order to investigate the association of sports facility accessibility with physical activity. Moreover, we conducted additional analyses to investigate the association between sports facility accessibility and physical activity according to history of depression and diverse sociodemographic groups based on age, income, occupation, and regional area.

## Methods

### Study participation

We analyzed data from the Community Health Survey (CHS), which has been conducted by the Korean Centers for Disease Control and Prevention (KCDC) since 2008, to set and evaluate regional health plans and produce comparable regional health statistics by standardizing the survey system. We used data available from the 2012 CHS, which collected information from a total of 228,921 people aged 20 years or older. We excluded individuals with data missing for sports facility accessibility (*n* = 1756), physical activity (*n* = 806), history of depression (*n* = 55), BMI (*n* = 12,852), household income (*n* = 11,202), and other variables (*n* = 527); therefore, a final sample population of 201,723 people was selected for this study. The CHS received consent from study participants before the beginning of the study. Instruments and study processes used for the survey were approved by the KCDC Institutional Review Board (IRB #: 2012-07CON-01-2C).

### Study variables

#### Sports facility accessibility

To evaluate sports facility accessibility, we utilized responses to the CHS question “During the past year, was it easy to find sports facilities near your house?” Sports facilities consist of not only the place where sports equipment is available, but also the exercise environment. We classified the answers “easy to find” and “very easy to find” as easy and “difficult to find” and “very difficult to find” as difficult.

#### Physical activity

Physical activity was investigated using the CHS questionnaire data, which comprised three types of answers: vigorous, moderate, and walking. The questionnaire also requested the number of days of each activity per week (i.e., “How many days did you perform vigorous physical activity that made you feel tired or breathless during the past week?) and minutes of activity per day (i.e., “For how many minutes did you perform vigorous physical activity during the day?”). ‘Vigorous’ was defined as activity burning at least 7/kcal per minute, including activities such as jogging, running, climbing, football, baseball, intensive aerobic activity, swimming, squash, and work activities requiring running. ‘Moderate’ activity included yoga, badminton, volleyball, and work activities using both the arms and legs. Based on these definitions, we used the International Physical Activity Questionnaire short forms (IPAQ) to classify the level of physical activity engaged in by each person in this study [23]. The IPAQ suggests a metabolic equivalent task (MET) for each level of physical activity as follows:

- Vigorous MET-minutes/week = 8.0 * minutes of activity per day * days of activity per week
- Moderate MET-minutes/week = 4.0 * minutes of activity per day * days of activity per week
- Walking MET-minutes/week = 3.3 * minutes of activity per day * days of activity per week

The IPAQ classified an individual’s activity as “Health-Enhancing Physical Activity (HEPA)” when the total score was 3000 MET-minutes (50 MET-hours)/week or more, “Active” when the total score was 600 MET-minutes (10 MET-hours) or more, and “Inactive” when the total score was below 600 MET-minutes (10 MET-hours). In this study, “HEPA” and “Active” were considered as participate, and “Inactive” was considered as non-participate [24].

#### Depression

To identify people with a history of depression, we utilized the response to the CHS question “Have you ever been diagnosed as depressed by doctor?” Answering alternatives were binary (yes/no).

#### Covariates

We used the covariates of sex, age (under 40, 40 to 64, 65 or over), educational level (elementary school, middle school, high school, and college or higher), marital status (unmarried, married-cohabiting, married-not-cohabiting), and regional area (urban and rural) as sociodemographic variables, and monthly income (classified by quartile) and occupation (white collar, pink collar, and blue collar) as economic variables. Finally, health variables, such as the amount of sleep (less than 7 h, 7 to 8 h, and 9 h or more), self-rated stress, perceived health status (good, normal, and bad), perceived body shape (thin, normal, and obese), current drinker (yes and no), current smoker (yes and no), and history of depression (yes and no) were used as covariates. Obesity was measured by Body Mass Index (BMI: weight (kg)/height (m) ^2^; no: BMI <25, yes: BMI ≥25). All covariates were treated as categorical variables.

### Statistical analyses

The purpose of this study was to analyze the factors affecting physical activity and the association of sports facility accessibility with physical activity. We performed statistical analyses of the survey data using SAS version 9.4 (SAS Institute, Cary, NC, USA). We first analyzed the distribution of each categorical variable described above to calculate the frequency and percentage of each variable and to identify significant differences between groups using the Chi-squared test. Next, we performed a multivariable logistic regression analysis to identify the relationship between sports facility accessibility and physical activity by controlling potential confounders including age, sex, monthly income, educational level, occupation, marital status, regional area, sleeping time, perceived stress rate, history of depression, perceived health status, current smoker, current drinker, perceived body shape, and obesity. Finally, we conducted subgroup analyses to investigate this association according to depression diagnosed experience, monthly house income, age, occupation, and regional area. The sampling weights were considered given that the CHS was a complex survey design. We produced adjusted odds ratios (ORs) with 95 % confidence intervals (95 % CIs).

## Results

The general characteristics for the 201,723 individuals participating in this study are listed in Table 1. Of the 201,723 study participants, 80.9 % (*n* = 155,331) felt that sports facilities were easily accessible, and 19.1 % (*n* = 46,392) reported participating in some type of physical activity. Of the total cohort, 2.2 % (*n* = 4918) had been diagnosed with depression. The cohort was largely represented by those holding blue-collar jobs, with 26.3 % (*n* = 40,023), 14.2 % (*n* = 26,515), and 59.5 % (*n* = 135,185) of the total cohort holding white-collar, pink-collar, and blue-collar occupations, respectively.

**Table 1:** General characteristics of the study participants

Variables	Total		Physical activity			
			No		Yes	
	*N*	(%)*	*N*	(%)*	*N*	(%)*
Accessibility to sports facilities						
Easy	155,331	80.9	96,524	50.4	58,807	30.5
Difficult	46,392	19.1	29,965	12.8	16,427	6.3
Sex						
Men	94,147	50.5	52,151	28.0	41,996	22.5
Women	107,576	49.5	74,338	35.2	33,238	14.4
Age group						
Under 40	57,187	39.4	36,956	25.2	20,231	14.1
40–65	103,312	48.7	60,439	29.1	42,873	19.6
65 or over	41,224	11.9	29,094	8.9	12,130	3.1
Monthly income						
Q1 (low)	53,233	16.1	35,515	11.2	17,718	4.9
Q2	50,304	24.4	31,829	15.9	18,475	8.5
Q3	55,050	31.2	33,869	19.5	21,181	11.7
Q4 (high)	43,136	28.2	25,276	16.5	17,860	11.7
Educational level						
Elementary school	49,443	13.6	33,091	9.8	16,352	3.9
Middle school	23,493	9.1	14,502	5.9	8,991	3.2
High school	71,301	39.6	43,805	24.5	27,496	15.0
College or over	57,486	37.7	35,091	23.0	22,395	14.7
Occupation						
White collar	40,023	26.3	24,794	16.2	15,229	10.1
Pink collar	26,515	14.2	16,497	8.8	10,018	5.4
Blue collar	135,185	59.5	85,198	38.1	49,987	21.4
Marital status						
Married-cohabit	143,384	67.3	87,791	42.2	55,593	25.2
Married-non cohabit	28,414	10.7	20,312	7.7	8,102	3.0
Unmarried	29,925	22.0	18,386	13.3	11,539	8.7
Regional area						
Urban	116,838	81.7	75,470	51.9	41,368	29.8
Rural	84,885	18.3	51,019	11.3	33,866	7.1
Sleeping time						
Less (<7)	87,607	44.8	54,400	27.9	33,207	16.9
Normal (7–8)	105,494	51.3	65,891	32.5	39,603	18.8
Exceed (> = 9)	8,622	3.8	6,198	2.7	2,424	1.1
Perceived stress rate						
Much	52,680	28.2	34,048	18.3	18,632	9.8
Little	109,873	55.7	67,814	34.7	42,059	21.0
Non	39,170	16.1	24,627	10.0	14,543	6.0
History of depression						
Yes	4,918	2.2	3,383	1.5	1,535	0.7
No	196,805	97.8	123,106	61.6	73,699	36.2
Perceived health status						
Good	80,996	44.2	46,660	25.4	34,336	18.7
Normal	82,126	41.9	52,291	27.5	29,835	14.4
Bad	38,601	14.0	27,538	10.2	11,063	3.7
Current smoker						
Yes	43,473	24.0	25,032	14.0	18,441	10.0
No	158,250	76.0	101,457	49.1	56,793	26.9
Current drinker						
Yes	135,821	74.6	81,146	45.1	54,675	29.5
No	65,902	25.4	45,343	18.1	20,559	7.4
Perceived body shape						
Thin	36,884	17.1	23,969	11.2	12,915	5.8
Normal	93,003	45.0	57,094	27.9	35,909	17.1
Obese	71,836	38.0	45,426	24.1	26,410	13.9
Obesity						
No (BMI <25)	153,502	76.0	97,354	48.7	56,148	27.3
Yes (BMI > =25)	48,221	24.0	29,135	14.5	19,086	9.5
	201,723	100.0	126,489	63.1	75,234	36.9

*(%): Weighted percentage

The odds ratios of factors associated with physical activity, and determined using a logistic regression analysis, are listed in Table 2. Those with easy access to sports facilities were 1.16 times more likely to participate in physical activity than those without easy access to sports facilities (OR = 1.16, 95 % CI 1.13–1.20). Less physical activity was also observed among low-income groups than among high-income groups. Those with white-collar occupations were less likely than those with blue-collar occupations to participate in physical activity. Those with less than 7 h of sleep per night were about as likely to participate in physical activity as those with 7–8 h of sleep per night, while those sleeping for 9 h or more each night were less likely to exercise. Finally, those with lower perceived health (normal or bad) exercised less than those with better-perceived health.

**Table 2:** Factors associated with physical activity*

Variables	Physical activity	
	Adjusted OR	95 % CI
Accessibility to sports facilities		
Easy	1.16	(1.13–1.20)
Difficult	1.00	-
Sex		
Men	1.91	(1.85–1.97)
Women	1.00	-
Age group		
Under 40	1.00	-
40–65	1.42	(1.38–1.47)
65 or over	0.93	(0.88–0.98)
Monthly income		
Q1 (low)	0.78	(0.75–0.82)
Q2	0.80	(0.77–0.83)
Q3	0.86	(0.83–0.89)
Q4 (high)	1.00	-
Educational level		
Elementary school	0.93	(0.88–0.97)
Middle school	0.93	(0.88–0.97)
High school	0.93	(0.90–0.96)
College or over	1.00	-
Occupation		
White collar	1.00	-
Pink collar	1.15	(1.10–1.21)
Blue collar	1.18	(1.14–1.22)
Marital status		
Married-cohabit	1.00	-
Married-non cohabit	0.92	(0.88–0.96)
Unmarried	1.21	(1.16–1.25)
Regional area		
Urban	1.00	-
Rural	1.23	-
Sleeping time		
Less (<7)	1.07	(1.05–1.10)
Normal (7–8)	1.00	-
Exceed (> = 9)	0.77	(0.72–0.82)
Perceived stress rate		
Much	0.94	(0.90–0.98)
Little	0.98	(0.94–1.01)
Non	1.00	-
History of depression		
Yes	1.14	(1.04–1.24)
No	1.00	-
Perceived health status		
Good	1.00	-
Normal	0.76	(0.74–0.78)
Bad	0.62	(0.59–0.65)
Current smoker		
Yes	0.87	(0.84–0.90)
No	1.00	-
Current drinker		
Yes	1.27	(1.23–1.31)
No	1.00	-
Perceived body shape		
Thin	1.00	-
Normal	1.16	(1.12–1.20)
Obese	1.10	(1.06–1.15)
Obesity		
No (BMI <25)	1.00	-
Yes (BMI > =25)	1.06	(1.02–1.10)

*The result of multivariable logistic regression to investigate the association between accessibility to sports facilities and physical activity controlling for sex, age, monthly income, educational level, occupation, marital status, regional area, sleeping time, perceived stress rate, history of depression, perceived health status, current smoker, current drinker, perceived body shape and obesity

The association between physical activity and access to sports facilities stratified by history of depression, monthly house income, age, occupation and regional area is shown in Table 3. The subgroup analysis showed significant differences in each group, although modifying effects were not significant in the tests for interaction except for occupation and regional area. Those who had experience of depression showed a trend towards a greater magnitude of physical activity if participants felt that they had easy access to sports facilities. There was also a trend towards a greater magnitude of physical activity if participants lived in an urban area (OR = 1.21, 95 % CI 1.16–1.26).

**Table 3:** Subgroup analysis of physical activity with accessibility to sports facilities stratified by history of depression, monthly income, age, occupation and regional area*

Variables	Physical activity		
	Difficult	Easy	
	OR	OR	95 % CI
History of depression			
Yes	1.00	1.46	(1.21–1.76)
No	1.00	1.16	(1.12–1.19)
Monthly income			
Q1 (low)	1.00	1.16	(1.09–1.23)
Q2	1.00	1.17	(1.10–1.24)
Q3	1.00	1.18	(1.11–1.25)
Q4 (high)	1.00	1.14	(1.07–1.23)
Age group			
Under 40	1.00	1.19	(1.13–1.26)
40–65	1.00	1.14	(1.09–1.19)
65 or over	1.00	1.15	(1.08–1.24)
Occupation			
White collar	1.00	1.24	(1.15–1.33)
Pink collar	1.00	1.14	(1.09–1.19)
Blue collar	1.00	1.15	(1.07–1.23)
Regional area			
Urban	1.00	1.21	(1.16–1.26)
Rural	1.00	1.06	(1.02–1.11)

*The result of subgroup analyses by using multivariable logistic regression to investigate the association between accessibility to sports facilities and physical activity controlling for sex, age, monthly income, educational level, occupation, marital status, regional area, sleeping time, perceived stress rate, history of depression, perceived health status, current smoker, current drinker, perceived body shape and obesity

## Discussion

In this study, we analyzed the factors associated with physical activity among people aged 20 or over, focusing on the population’s access to sports facilities. Our observations show that sports facility accessibility is considerably associated with the amount of physical activity an individual participates in.

Previous work suggested that the distance required of an individual to travel to a sports facility affects sports facility usage [14, 17, 18], as in the present study. This association may be explained by the fact that environmental factors affect an individual’s perception of physical activity [25]. Long distances may reduce the motivation to do physical activity [26], as inability to access appropriate facilities is reported to likely act as perceived motivational barrier [27]. Therefore, easy access to sports facilities may act as a motivator to encourage an individual to participate in physical activity [28]. For example, an individual living near a sports facility can easily access information about exercise, while those living further away from sports facilities cannot easily access this information [25]. Another study reported similar results, clarifying that an individual’s physical environment should be treated as a subsidiary determinant factor because it does not affect the frequency of physical activity as much as other factors including social support [29].

In addition, our subgroup analysis indicated that history of depression, monthly household income, age, occupation, and regional area potentially affect the association between access to sports facilities and physical activity, although the modifying effects were not significant. The results of the present study showed that easy access to sports facilities among those who had a history of depression tended to result in more physical activity than when such facilities were less accessible due to distance. Generally, depressed people have insufficient motivation to maintain an active lifestyle [30]. However, taking into account the various health and well-being benefits proffered by physical activity, it is important to encourage depressed individuals to exercise. According to a previous study, there is a clear association between autonomous types of motivation and physical activity [31]; therefore, proximity to sports facilities may encourage depressed individuals to spontaneously participate in physical activity by increasing opportunities to acquire information about exercise and access to sports equipment. The proximity of sports facilities was associated with physical activity regardless of monthly household income, age, and occupation. Regardless of monthly household income, easier access to sports facilities was associated with increased physical activity; therefore, although low income populations are usually at risk for physical inactivity [32], access to sports facilities appears to more strongly affect physical activity than does income, with easy access to sports facilities promoting physical activity even among those at the highest risk for inactivity [33].

We also observed an association between an individual’s occupation and physical activity. Without taking sports facility accessibility into account, those classified as white-collar workers exercise less than blue-collar workers. Because white-collar jobs are typically sedentary, the amount of physical activity experienced on the job by the white-collar worker is less than that experienced by the blue-collar worker, who typically does some type of physical activity while working [34, 35]. However, according to this study, when access to sports facilities was higher, white-collar workers showed higher rate of physical activity. A previous study suggested that promoting physical activity in the workplace, such as facilitating access to sports facilities, increases exercise among workers [36], reducing barriers to physical activity and promoting physical activity regardless of the risk for inactivity.

The current findings should be interpreted with a degree of caution due to several limitations. First, sports facility accessibility was assessed via a single self-reported questionnaire. Therefore, we could only acquire the perceived, rather than actual, distance between an individual’s home and sports facilities. Moreover, there was the possibility of same-source bias, as the exposure and outcome variables were self-reported. Additionally, objective accessibility could not be estimated in this study. It was also difficult to identify whether participants were depressed at the time that they took the survey, as the survey only asked whether a person had ever been diagnosed with depression and did not specify whether the diagnoses was current. Moreover, this study could only be generalizable to South Korean adults. In addition, there was potential confounding influence from other area-level factors (i.e., the proximity to a sports club may be associated with other area-level factors that are associated with physical activity). Furthermore, the measurement of physical activity might have been inaccurate due to the validity of the IPAQ, which is known to overestimate physical activity relative to objectively measured data in most populations [37]. Therefore, the physical activity levels used in this study might have been overestimated. Finally, it should be noted that we used cross-sectional data; therefore, we could not exclude a bi-directional effect or relationship opposite to what was hypothesized (i.e., those who are more physically active self-select into areas with a higher density of sports clubs).

Despite these limitations, this study also has several strengths. First, compared to previous studies that set a limit on particular generations, this study utilized data from adults aged 20 years to 65+ and included a cohort of those with a history of depression diagnosis. To our knowledge, this is the first attempt to investigate the association between sports facility accessibility and physical activity among those with a history of depression. Secondly, considering that perceived barriers to health-promoting behaviors are an important component of major health behavior theories [24], this study provides useful data by implementing individuals’ perceived level of access to sports facilities. Finally, we used national sample data, suggesting that our data can be widely generalized.

Our results suggest that an individual’s perceived level of access to sports facilities may play an important role in physical activity of not only the general population, but also of those with a history of depression. Therefore, it is crucial to take into account sports facility accessibility when building physical activity-promoting environments or designing programs for enhancing physical activity. Additionally, this study provides a basis for future research on treating depression through physical activity. Based on these observations, further studies should investigate the association between physical activity and sports facility accessibility among cohorts with other conditions that improve with physical activity. Future studies should also use more concrete methods for investigating sports facility accessibility, such as by using both a geographic information system for investigating objective accessibility and several questionnaires rather than only one questionnaire for investigating subjective accessibility.

## Conclusion

Our results showed that the accessibility of sports facilities is associated with physical activity. Therefore, it is crucial to consider the accessibility of sports facilities when promoting an environment conducive to physical activity or designing programs for enhancing physical activity.

## Abbreviations

CHS: Community health survey
HEPA: Health-enhancing physical activity
IPAQ: International physical activity questionnaire
KCDC: Korean centers for disease control and prevention
MET: Metabolic equivalent task
